# A Systematic Review of Post-COVID Electrocardiographic Changes in Young Athletes

**DOI:** 10.7759/cureus.31829

**Published:** 2022-11-23

**Authors:** Thiago A Laranjeira, Antonio S Menezes

**Affiliations:** 1 Medicine, Pontifical Catholic University of Goiás, Goiânia, BRA; 2 Cardiology, Pontifical Catholic University of Goiás, Goiânia, BRA; 3 Internal Medicine/Cardiology, Federal University of Goiás, Goiânia, BRA

**Keywords:** systematic review, long covid-19, cardiology research, young athletes, ecg (electrocardiogram), covid-19

## Abstract

Post-coronavirus disease (COVID) syndrome involves the presentation of various new, returning, or ongoing symptoms, more than four weeks after COVID-19 infection. Post-infectious myocarditis is a potential sequela, associated with greater arrhythmogenic potential. Thus, it is an outcome that should be considered in athletes. An undiagnosed heart condition associated with adrenergic stimulus caused by high-intensity exercises can lead to sudden cardiac death. Electrocardiography (ECG) plays a role in cardiac screening for potential cardiac changes associated with myocarditis. Therefore, this study aimed to evaluate the occurrence of electrocardiographic alterations in athletes during the post-COVID period.

A systematic review of longitudinal observational studies in the PubMed, LILACS, and CENTRAL databases that evaluated athletes in the post-COVID period with ECG was performed. A total of four articles involving 5371 patients were included in the analysis. The athletes mostly presented with mild asymptomatic or symptomatic COVID-19. A low prevalence of electrocardiographic alterations suggestive of cardiac involvement by severe acute respiratory syndrome coronavirus 2 (SARS-CoV-2) was identified in this population.

Electrocardiographic abnormalities indicative of myocarditis are uncommon in young athletes throughout the post-COVID era. However, anomalies that may signify and need further cardiovascular testing were found.

## Introduction and background

The coronavirus disease 2019 (COVID-19) pandemic has resulted in the development of post-COVID syndrome (PCS). PCS occurs in individuals with confirmed or probable severe acute respiratory syndrome coronavirus 2 (SARS-CoV-2) infection, at least three months after disease onset, with a symptom duration of at least two months, and where the symptomatology cannot be explained by another diagnosis. Symptoms include fatigue, dyspnea, and cognitive changes, which may be intermittent or persistent [1]. Myocarditis is a late sequela associated with post-COVID syndrome since SARS-CoV-2 can directly infect myocardial cells, damaging the myocardium, and thus increasing the risk of malignant ventricular arrhythmias due to chronic residual healing [2].

In athletes, myocarditis secondary to viral infection is alarming due to the degree of adrenergic and hemodynamic stimulation caused by high-intensity exercise. This increases the arrhythmogenic potential of heart disease [3]. However, an athlete’s heart may present adaptive changes to exertion, which can be confused with pathological findings. Therefore, the correct interpretation of such findings is key [4].

Regardless of PCS, electrocardiographic evaluation is one of the key aspects of the pre-participation evaluation of athletes since several disorders associated with sudden cardiac death (SCD) of athletes can be identified by electrocardiogram (ECG). Previous SARS-CoV-2 infection can be identified by observing T-wave inversion and ST-segment abnormalities since, in 33% of cases, such changes are associated with myocardial inflammation [5].

In this sense, the cardiovascular evaluation of athletes is important in the context of PCS. Moreover, ECG findings should be correctly interpreted to differentiate physiological cardiac changes associated with regular and intense exercise and features suggestive of heart disease, as recommended by the International Criteria for interpretation of the athlete's ECG [6]. Furthermore, electrocardiographic alterations found following SARS-CoV-2 infection should be investigated due to the arrhythmogenic potential of myocarditis in athletes.

An uncertain risk of myocarditis exists in young athletes; therefore, pre-participation screening to identify athletes who may require additional testing and medical care before returning to sports is essential. As the rate of myocarditis related to abnormal ST or T waves may be high, additional cardiac testing is essential. All athletes with such findings should be placed under permanent observation, as even asymptomatic myocarditis can result in permanent cardiac damage and sudden cardiac death [7].

The following aspects of ECG should be considered to prevent sudden cardiac death in young athletes: first, additional cardiac examinations should be performed in cases where abnormalities in the P wave, QRS complex, ST segment, T waves, QT interval, cardiac rhythm, and conduction are present [7-9]. Second, in addition to using an ECG in pre-participation examinations to screen for rare genetic and congenital conditions, checking for cardiovascular sequelae of COVID-19 is also important. This enables the detection of patterns that are indicative of myocardial inflammation such as T-wave inversions and new ST-segment changes [7-11]. Subsequent endomyocardial biopsy results indicated histological evidence of myocarditis in 33% of all cases in which aberrant ST or T waves were detected [11]. Therefore, this study aimed to evaluate the occurrence of electrocardiographic alterations in athletes during the post-COVID period.

## Review

Materials and methods

This study was a systematic review based on the Preferred Reporting Items for Systematic Reviews and Meta-Analyses (PRISMA) statement. Articles were obtained by searching the PubMed (36 articles), LILACS (1 article), and CENTRAL (4 articles) online databases for articles from the last two years. Articles were selected based on previous knowledge of the authors in the literature on the subject under investigation. Articles with the descriptors "COVID-19" "Athletes" AND "Electrocardiogram" were included. Our search description: (("COVID-19" or "Long Covid-19" or "Late Covid-19") AND (" Athletes") AND ("Electrocardiogram")).

Two researchers evaluated the articles separately and independently, first reading the article titles and abstracts to verify their eligibility for the theme addressed in this study. Articles that could be included were read in full by the researchers and included or excluded based on their characteristics. There were no limitations regarding the language of the studies, sex, or age of the participants. Longitudinal observational studies that evaluated athletes after the resolution of COVID-19 were included to determine the incidence of electrocardiographic changes in athletes previously infected with SARS-CoV-2. In the first database search, 41 articles were obtained, of which 12 were selected for full reading and 4 met the inclusion criteria of our study.

Results

**Figure 1:**
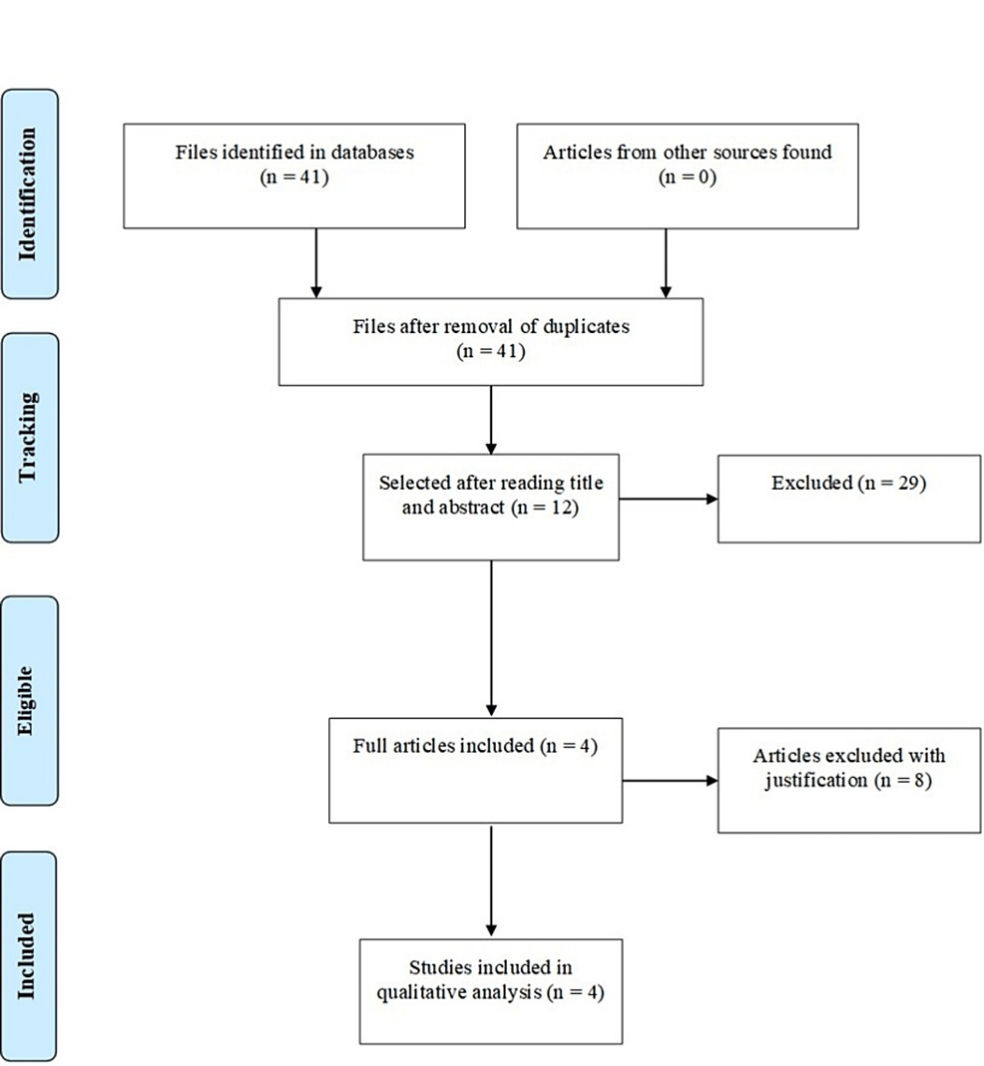
Study selection strategy

In total, four studies were included in the integrative review, as seen in Figure 1. The selected studies were conducted between 2021 and 2022, and the sample size ranged from 99 to 4313, totaling 5371 athletes who underwent ECG evaluation after the resolution of COVID-19. The characteristics of these studies and the profile of previous SARS-CoV-2 infections in athletes are described in Table 1 and Table 2, respectively.

**Table 1:** Characteristics of study participants SD: standard deviation

First author	Type of study	Year of publication	Number of participants	Sex		Age average (SD)
				Male	Female	
Erickson et al. [7]	Retrospective cohort	2021	170	91 (53.5%)	79 (46.5%)	19.5 (± 1.5)
Martinez et al. [8]	Retrospective cohort	2021	789	777 (98.5%)	12 (1.5%)	25.0 (± 3.0)
Guevarra et al. [9]	Retrospective cohort	2022	99	68 (68.7%)	31 (31.3%)	19.9 (± 1.7)
Casasco et al. [10]	Prospective cohort	2022	4313	2810 (67.8%)	1333 (32.2%)	22.5 (± 13.3)

**Table 2:** Summary of previous infections by SARS-CoV-2

Study	Infection profile			
	Asymptomatic	Mild	Moderate	Serious
Erickson et al. [7]	116 (68.2%)	31 (18.2%)	1 (0.5%)	6 (3.5%)
Martinez et al. [8]	329 (41.7%)	460 (58.3%)	0 (0.0%)	0 (0.0%)
Guevarra et al. [9]	31 (31.3%)	51 (51.5%)	17 (17.2%)	0 (0.0%)
Casasco et al. [10]	2168 (52.3%)	1924 (46.4%)	44 (1.1%)	6 (0.2%)

Four studies used the international criteria for the interpretation of the athlete ECGs to categorize the findings as normal or abnormal. Erickson et al. identified six athletes (3.5%) out of 170 with abnormal or borderline electrocardiographic alterations, and they were subsequently referred for cardiovascular evaluation [7]. The severity of symptoms during the SARS-CoV-2 infection period was not associated with a higher risk of ECG abnormalities (χ2 = 0.054; p = 0.817). Moreover, there was no statistically significant association between sex, body mass, and electrocardiographic alterations. The ECG abnormalities found by Erickson et al. are listed in Table 3 [7]. Subsequent evaluation of the six athletes by echocardiography, troponin dosage, and cardiac magnetic resonance imaging (CMR) did not reveal heart disease in five of the athletes; however, one athlete (16.6%) was diagnosed with effusive viral pericarditis.

**Table 3:** Electrocardiographic abnormalities in the study by Erickson et al. Source: [7]

Patient	Abnormal ECG result
1	Sinus bradycardia with septal infarction of undetermined age
2	Sinus bradycardia with T-wave abnormality; consider anterior ischemia
3	Sinus bradycardia with nonspecific ST-segment abnormality
4	Sinus bradycardia with right branch block (BRD)
5	Sinus bradycardia with T-wave abnormality; consider lower ischemia
6	Sinus bradycardia with nonspecific ST-segment abnormality

Martinez *et *al. studied 789 elite athletes who tested positive for COVID-19 before resuming team sports [8]. Abnormal screening test findings led to cardiac magnetic resonance imaging or stress echocardiography. After the cardiac screening, professional athletes had no cardiac incidents. After a positive test, testing took 19 (17) days (range, 3-156 days). Thirty athletes (3.8%; troponin, 6 athletes (0.8%); ECG, 10 athletes (1.3%); echocardiography, 20 athletes (2.5%)) had abnormal screening results, requiring additional testing; 5 athletes (0.6%) had cardiac magnetic resonance imaging findings suggesting inflammatory heart disease (myocarditis, 3; pericarditis, 2) that restricted play.

Two individuals (2.0%) out of 99 tested by Guevarra et al. had abnormal results that were consistent with post-infectious cardiovascular dysfunction [9]. There was a case of V1-V4 T wave inversion in one athlete, and a diagnosis of recurrent unifocal premature ventricular contractions in another. Following further investigation, the troponin levels in both athletes were normal, and their CMR was unaltered. There was no correlation between the intensity of COVID-19 symptoms and either sex or ECG changes. One athlete showed abnormal ECG readings before starting the sports season; however, exercise treadmill stress testing indicated numerous premature ventricular complexes that disappeared with activity. After considering the abnormal pre-season ECG findings, the incidence of aberrant ECG values during infection was 1%, and no additional signs of myocarditis were identified in this case. All other cardiac tests in this group of athletes, for their return to sport, were also within normal limits.

In an electrocardiographic study by Casasco et al. arrhythmic events were found [10]. In total, 91 athletes (2.20%) were found to have ventricular arrhythmias, the majority of which were composed of single premature ventricular beats (PVBs) and pairs of PVBs. Seven cases of bigeminy, three cases of trigeminy, and 12 cases of PVBs were found among these athletes. One athlete had a series of PVBs while exercising, but these were not accompanied by any warning signs. The incidence of PVBs was ranked according to the athletes’ clinical presentation severity. During 24-hour observation, ECG revealed arrhythmic episodes in a further 10 athletes (0.24%).

Discussion

The correct interpretation of ECG of athletes becomes important in the context of the post-COVID syndrome since physiological findings associated with regular practice of high-intensity exercises and alterations that increase the risk of sudden cardiac death (SCD) in this population should be differentiated. Therefore, international criteria define normal, borderline, and abnormal findings for the interpretation of athlete ECGs. The presence of at least two borderline or one abnormal finding warrants further cardiovascular evaluation. The association of alterations, such as T-wave inversion and ventricular arrhythmias, with myocarditis, is well-documented as a possible sequela of SARS-CoV-2 infection [6].

Before the COVID-19 pandemic, the isolated use of 12-lead ECG for the diagnosis of myocarditis had a sensitivity of 47% and an unknown specificity. Moreover, ECG abnormalities were mostly found in sinus tachycardia with nonspecific alterations of the T-wave [11]. However, ECG abnormalities associated with alterations in other cardiac tests, in particular, the development of symptoms after returning to sports warrant further cardiovascular assessment. Such findings should not be interpreted by an ECG alone [12]. The case for the implementation of ECG in the pre-participation evaluation of sports can be supported. One study showed an 84% reduction in cases of SCD following the implementation of an ECG screening program for individuals between 12 and 35 years of age [13].

The results of this review support those of the existing literature, suggesting that ECG readings of high-performance athletes have low specificity, especially where ECG abnormalities occur in the absence of other cardiovascular examination findings. One study demonstrated that even in the absence of cardiovascular alterations, 9% of athletes in the study cohort had late enhancement with gadolinium on CMR, and half of these athletes met the criteria for myocarditis associated with previous SARS-CoV-2 infection [14].

Observing the possible symptoms associated with the post-COVID syndrome is also necessary to indicate cardiovascular evaluation after ECG. In a study that evaluated 137 athletes, it was demonstrated that only one athlete presented with ECG abnormalities. However, five athletes were diagnosed with cardiac sequelae associated with SARS-CoV-2 infection. Moreover, 21% of athletes with chest pain induced by exercise after returning to sports had cardiac involvement in CMR, possibly related to COVID-19 [15].

The adoption of ECG as an isolated strategy for the evaluation of sports pre-participation after the resolution of COVID-19 should not be recommended, despite being a low-cost alternative. This is due to ECG being ineffective in detecting possible cardiac diseases in athletes with mild asymptomatic or symptomatic previous SARS-CoV-2 infection. However, ECG may represent a valuable strategy for initial screening and indications for further cardiac evaluation. In a study of 19,378 athletes, cardiac involvement was detected more frequently with CMR, in patients who underwent CMR following preliminary ECG, troponin, and transthoracic echocardiogram, than in athletes who only underwent primary CMR [16].

The low lethality of COVID-19 among individuals aged 15 to 24 years associated with preliminary findings, indicates that cardiac involvement of COVID-19 is rare and that the low incidence of ECG alterations in the post-COVID study suggests the risk for young athletes is low. However, ignorance regarding the long-term effects of SARS-CoV-2 infection supports pre-participation sports evaluation, especially for athletes with persistent symptoms after the resolution of the acute viral condition. Moreover, athletes should take precautions to prevent initial SARS-CoV-2 infection and, if infected, resume activities with caution and monitor the persistence of symptoms [17].

The Brazilian Guidelines for Sports Cardiology, recommend a resting 12-lead ECG for pre-participation screening (PPS) of both amateur and professional athletes. This enables the identification of any possible changes correlating with previously mentioned diseases as the most common causes of SCD [18]. Alterations that may be associated with pericarditis or myocarditis should be observed in people who have been exposed to COVID-19. These changes include ST-segment variations (typically ST-segment depression), T-wave inversion, conduction abnormalities (including full left bundle branch block and atrioventricular blocks), and complex supraventricular and ventricular arrhythmias [19].

Findings of an Italian study showed 26% of patients hospitalized with COVID-19 linked with pneumonia developed new ECG abnormalities within 51 days (mean, 20-30 days) of symptom onset when compared to their admission ECGs. The most frequent ECG abnormalities were bradycardia (2%), atrial fibrillation (6%), and persistent ST alterations (14%). Higher levels of troponin were observed in 38% of patients. However, these alterations did not correspond with the severity of pulmonary symptoms, occasionally manifested on the eve of hospital discharge, and after a recent reverse transcription-polymerase chain reaction (RT-PCR) test returned negative results [20].

As physiological changes occur in the heart as a direct result of physical activity, it is essential to emphasize that physically fit people and athletes often exhibit a distinctive ECG pattern compared to that of the general population. Considering this, ECG interpretation should preferably be performed by a cardiologist with previous experience in sports, and by the most recent international recommendations for the interpretation of electrocardiographic studies in athletes [21]. Furthermore, comparing an ECG obtained after COVID-19 with an ECG obtained from an athlete before COVID-19 is particularly helpful. Any new alterations should be considered suspicious and should warrant further cardiac testing.

Šarčevićet al. reported an increased number of ECG findings requiring further additional cardiac examinations in young athletes during the coronavirus [22]. The study reported that the number of ECG findings requiring additional cardiac examination, according to modern sports cardiology, was significantly higher in 2020 than in the three previous years.

A case-control study investigated the effects of asymptomatic and mild COVID-19 on transmyocardial repolarization parameters in children without treatment [17]. The findings showed that the QTd, QTcd, Tp-e, Tp-e dispersion, Tp-e/QT ratio, and Tp-e/QTc ratio were significantly higher in the COVID-19 group than in the control group. Moreover, ventricular repolarization was impaired in children with asymptomatic COVID-19 [17].

Furthermore, another study [14] described the association between COVID-19 and T-wave inversion in a large case series of older patients (mean age, 66 ± 7 years) [22]. Thus, our study indicates the possibility that T-wave inversion may be a manifestation of COVID-19 in young athletes.

Limitations

Regarding the methodological limitations of the articles included in this systematic review, it is necessary to highlight that they corresponded to longitudinal observational studies, which do not present the highest quality level of evidence, with reduced sampling and the absence of a control group for comparison purposes. Moreover, because COVID-19 is a recent disease, the number of articles found in the databases that associated the condition with electrocardiographic alterations was reduced.

## Conclusions

Athletes in the post-COVID group had a low prevalence of ECG alterations suggestive of myocarditis, indicating that cardiac complications associated with a previous SARS-CoV-2 infection in this population are rare. However, ECG evaluation may provide clinical indications for abnormalities that warrant further subsequent cardiac evaluations with more sensitive and specific tests. This is especially important for athletes with persistent symptoms following the resolution of acute viral infections.

Further experimental studies are needed for the real evaluation of electrocardiographic alterations in athletes with symptoms characteristic of post-COVID syndrome. Due to the different methods of data collection and analysis used in the studies, it is necessary to establish a protocol that guides pre-participation sports evaluation for athletes with suspected cardiovascular findings with a history of previous SARS-CoV-2 infection, allowing further research on the subject and evaluating the population linearly.
